# Heterozygous lamin B1 and lamin B2 variants cause primary microcephaly and define a novel laminopathy

**DOI:** 10.1038/s41436-020-00980-3

**Published:** 2020-10-09

**Authors:** David A. Parry, Carol-Anne Martin, Philip Greene, Joseph A. Marsh, Genomics England Research Consortium, J. C. Ambrose, P. Arumugam, E. L. Baple, M. Bleda, F. Boardman-Pretty, J. M. Boissiere, C. R. Boustred, H. Brittain, M. J. Caulfield, G. C. Chan, C. E. H. Craig, L. C. Daugherty, A. de Burca, A. Devereau, G. Elgar, R. E. Foulger, T. Fowler, P. Furió-Tarí, A. Giess, J. M. Hackett, D. Halai, A. Hamblin, S. Henderson, J. E. Holman, T. J. P. Hubbard, K. Ibáñez, R. Jackson, L. J. Jones, D. Kasperaviciute, M. Kayikci, A. Kousathanas, L. Lahnstein, K. Lawson, S. E. A. Leigh, I. U. S. Leong, F. J. Lopez, F. Maleady-Crowe, J. Mason, E. M. McDonagh, L. Moutsianas, M. Mueller, N. Murugaesu, A. C. Need, C. A. Odhams, A. Orioli, C. Patch, D. Perez-Gil, M. B. Pereira, D. Polychronopoulos, J. Pullinger, T. Rahim, A. Rendon, P. Riesgo-Ferreiro, T. Rogers, M. Ryten, K. Savage, K. Sawant, R. H. Scott, A. Siddiq, A. Sieghart, D. Smedley, K. R. Smith, S. C. Smith, A. Sosinsky, W. Spooner, H. E. Stevens, A. Stuckey, R. Sultana, M. Tanguy, E. R. A. Thomas, S. R. Thompson, C. Tregidgo, A. Tucci, E. Walsh, S. A. Watters, M. J. Welland, E. Williams, K. Witkowska, S. M. Wood, M. Zarowiecki, Moira Blyth, Helen Cox, Deirdre Donnelly, Lynn Greenhalgh, Stephanie Greville-Heygate, Victoria Harrison, Katherine Lachlan, Caoimhe McKenna, Alan J. Quigley, Gillian Rea, Lisa Robertson, Mohnish Suri, Andrew P. Jackson

**Affiliations:** 1 MRC Human Genetics Unit, Institute of Genetics and Molecular Medicine, University of Edinburgh, Edinburgh, UK; 2 Yorkshire Regional Genetics Service, Leeds Teaching Hospitals NHS Trust, Department of Clinical Genetics, Chapel Allerton Hospital, Leeds, UK; 3 West Midlands Regional Genetics Service, Birmingham Women’s NHS Foundation Trust, Birmingham Women’s Hospital, Edgbaston, Birmingham, UK; 4 Northern Ireland Regional Genetics Service, Belfast City Hospital, Belfast, UK; 5 Liverpool Centre for Genomic Medicine, Liverpool Women’s Hospital, Liverpool, UK; 6 Faculty of Medicine, University of Southampton, Southampton, UK; 7 Wessex Clinical Genetics Service, University Hospital Southampton, University Hospital Southampton NHS Foundation Trust, Southampton, UK; 8 Wessex Clinical Genetics Service, Princess Anne Hospital, University Hospital Southampton NHS Foundation Trust, Southampton, UK; 9 Human Development and Health, Faculty of Medicine, University of Southampton, Southampton, UK; 10 Department of Radiology, Royal Hospital for Sick Children, Edinburgh, UK; 11 Department of Clinical Genetics, Aberdeen Royal Infirmary, Scotland, UK; 12 Clinical Genetics Service, Nottingham University Hospitals NHS Trust, City Hospital Campus, Nottingham, UK; 13 Genomics England, London, UK; 14 William Harvey Research Institute, Queen Mary University of London, London, UK

**Keywords:** *LMNB1*, *LMNB2*, laminopathy, primary microcephaly, neurodevelopmental disorder

## Abstract

**Purpose:**

Lamins are the major component of nuclear lamina, maintaining structural integrity of the nucleus. Lamin A/C variants are well established to cause a spectrum of disorders ranging from myopathies to progeria, termed laminopathies. Phenotypes resulting from variants in *LMNB1* and *LMNB2* have been much less clearly defined.

**Methods:**

We investigated exome and genome sequencing from the Deciphering Developmental Disorders Study and the 100,000 Genomes Project to identify novel microcephaly genes.

**Results:**

Starting from a cohort of patients with extreme microcephaly, 13 individuals with heterozygous variants in the two human B-type lamins were identified. Recurrent variants were established to be de novo in nine cases and shown to affect highly conserved residues within the lamin ɑ-helical rod domain, likely disrupting interactions required for higher-order assembly of lamin filaments.

**Conclusion:**

We identify dominant pathogenic variants in *LMNB1* and *LMNB2* as a genetic cause of primary microcephaly, implicating a major structural component of the nuclear envelope in its etiology and defining a new form of laminopathy. The distinct nature of this lamin B–associated phenotype highlights the strikingly different developmental requirements for lamin paralogs and suggests a novel mechanism for primary microcephaly warranting future investigation.

## INTRODUCTION

The nuclear lamina is a protein structure that lines the inner nuclear membrane and provides structural support to the nucleus.^1^ Lamins are the major component of the nuclear lamina, forming a meshwork of filaments; they interact with numerous proteins and also act as a signaling hub, linking the nuclear lamina to the cytoskeleton and chromatin. Consequently, as well as maintaining structural integrity of the nucleus, they influence chromatin organization, DNA transcription, repair, and replication.^1^

Vertebrate cells express two classes of lamins, A and B, grouped based on sequence homology. Lamins A and C (A-types) are splice isoforms encoded by the same gene, while the B-type lamins are the products of different genes.^1^

Over the past two decades many disorders have been linked to *LMNA* variants, collectively termed laminopathies. Four major disease categories have been described with overlapping features: striated muscle diseases, lipodystrophy syndromes, peripheral neuropathies, and accelerated aging (segmental progeroid) disorders.^2^

In contrast to *LMNA*, few reports have associated human disease with variants in B-type lamin genes. No pathogenic single-nucleotide variants in *LMNB1* have been reported, although genomic duplications incorporating *LMNB1* cause adult-onset leukodystrophy (MIM 169500).^3^ For *LMNB2*, a homozygous missense variant in a family with progressive myoclonic epilepsy and ataxia has been described.^4^ Enrichment of heterozygous *LMNB2* variants in acquired partial lipodystrophy patients has also been reported.^5^

Microcephaly is a reduction in head size, defined by a head circumference at least three standard deviations below the mean for age and gender. The underlying reduction in brain volume has many etiologies: chromosomal, environmental, as well as single-gene syndromic and nonsyndromic causes. Primary microcephaly represents a recognizable monogenic form of microcephaly in which brain size is markedly reduced in the absence of other malformations and/or significant neurological deficits aside from intellectual disability.^6^ Many genes associated with centrosome and/or mitotic spindle function have been described, suggesting primary microcephaly to be a disorder of neural stem cell mitosis.^7^

Here we report the identification of recurrent heterozygous variants in *LMNB1* and *LMNB2*, implicating the nuclear lamina in the etiology of microcephaly.

## MATERIALS AND METHODS

### Ethics statement

Ethical approval was obtained as follows: Scottish Multicentre Research Ethics Committee (05/MRE00/74); Deciphering Developmental Disorders (DDD) Study (10/H0305/83, Cambridge South REC; GEN/284/12 Republic of Ireland REC); 100,000 Genomes Project (100kGP), East of England–Cambridge South REC (14/EE/1112). Informed written consent obtained for all participating families. Parents provided written authorization for publication of clinical photographs.

### Bioinformatics

DDD Study exome sequencing and variant calling were performed as previously described.^8^ De novo variants were identified using VASE (v0.2.4, https://github.com/david-a-parry/vase). For 100kGP, variants and Human Phenotype Ontology (HPO) terms were extracted from the Genomics England research environment.

For de novo variant calling criteria, structural modeling, and immunofluorescence experiment methodology, see Supplementary methods.

## RESULTS

### Identification of *LMNB1* pathogenic variants

To identify novel microcephaly genes, DDD exome sequencing data were analyzed in a subset of 1056 trios and singletons where the proband had microcephaly (occipital frontal circumference [OFC] <−4 SD below age and sex-matched mean; ~43% cases primary, 57% secondary microcephaly). This identified two individuals (P1 and P2) with de novo variants in *LMNB1* (Table S1, S2) resulting in substitution or deletion of the same amino acid residue (NM_005573.4:c.97A>G, p.Lys33Glu and c.97_99del, p.Lys33del). P3 was also heterozygous for the same c.97_99del variant but not confirmed de novo as the family was lost to follow up. In the 100kGP cohort further variants in *LMNB1* were identified in microcephalic patients, with P9 and P11 also having the same de novo c.97A>G variant as P1 (Table S3). An additional recurrent substitution (c.269G>C, p.Arg90Pro) was identified in P10 and P13. This was confirmed to be de novo in P10; however, no parental data were available for P13.

**Fig. 1:**
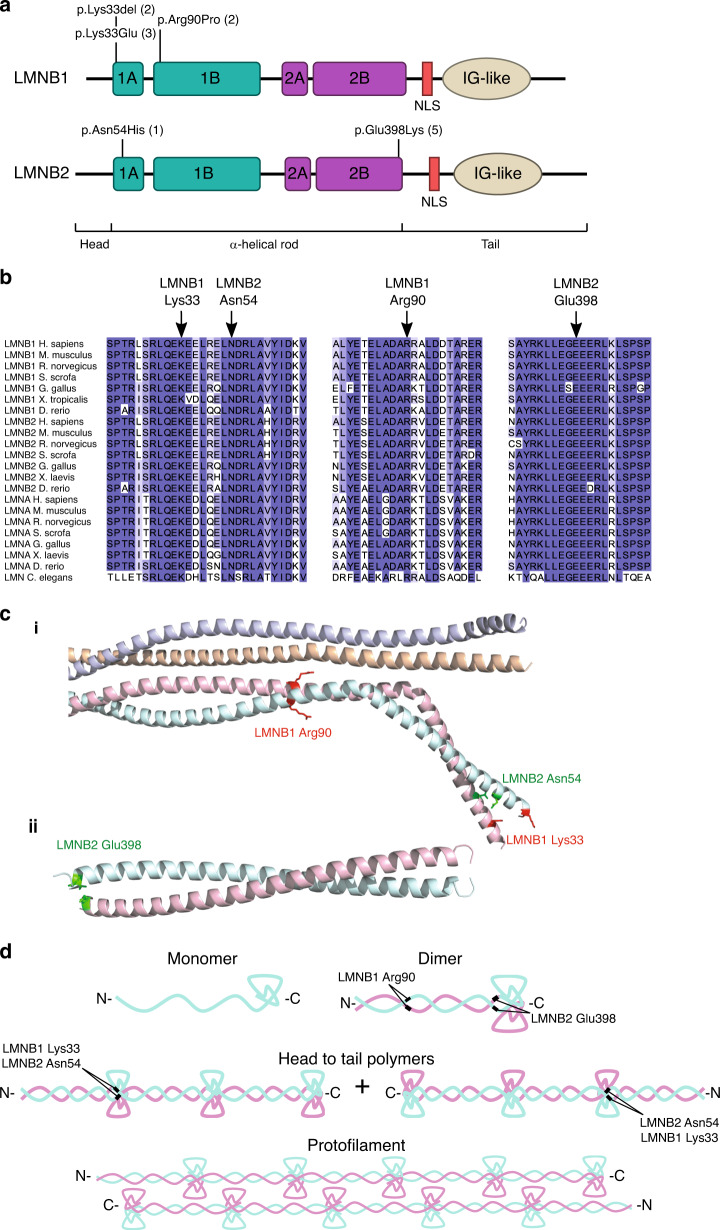
Pathogenic *LMNB1* and *LMNB2* variants occur recurrently at highly conserved residues at intra- and interdimer filament interfaces. (**a**) Schematic of B-type lamin protein structure. Locations of variants identified in microcephaly patients indicated, number of occurrences in parentheses. The ɑ-helical rod comprises coiled-coil domains 1A, 1B and 2A, 2C, indicated by green and purple boxes respectively. *IG-like*, immunoglobulin-like domain; *NLS,* nuclear localization signal. (**b**) Variants alter residues conserved in all vertebrate and invertebrate lamins. Lamins are specific to metazoa and the number of lamin genes has increased during evolution, with a single lamin gene in *C. elegans* compared with three mammalian lamin genes. Orthologs of both A- and B-type lamins aligned using Clustal X and colored by percent identity using Jalview. (**c**) Location of altered residues within lamin structures. While LMNB1/2 structures covering the mutated residues have not been published, as all residues are conserved with LMNA, we could use crystal structures of LMNA with Protein Data Bank (PDB) accessions 6JLB (i) and 1X8Y (ii) for molecular modeling and visualization. The upper structures shows a LMNA homotetramer, with the mutated residues highlighted on one of the constituent homodimers, while the bottom structure is of a LMNA homodimer, as no higher-order structure is available for this region. (**d**) Lamin filaments are assembled in a hierarchical fashion. First, lamin proteins form dimers, which further assemble into head to tail polymers. These polymers then laterally assemble in an antiparallel fashion to form lamin protofilaments.

In total, three recurrent *LMNB1* variants were identified in seven individuals (Fig. 1a), none of which were reported in gnomAD (v2.1). All were at residues conserved in all lamin metazoan homologs (Fig. 1b) and predicted damaging by multiple in silico tools (Table S4).

### *LMNB2* variants associated with microcephaly

Following identification of variants in *LMNB1*, we assessed whether variants in its close homolog *LMNB2* were also associated with microcephaly. We identified four microcephalic individuals from the DDD cohort (P4, P5, P6, P7) and one individual from the 100kGP study (P12) with the same variant in *LMNB2* (NM_032737.4: c.1192G>A, p.Glu398Lys) (Tables S1–S3). For cases P4, P5, and P12, the variant was established to be de novo, while in P7, it had been inherited from the proband’s mosaic, clinically unaffected, mother (Fig. S1). Finally, another case was identified with a distinct missense de novo variant (c.160A>C, p.Asn54His). These variants were absent from gnomAD (v2.1), at highly conserved residues (Fig. 1b) and predicted damaging (Table S4).

In total, in 13 microcephalic individuals we identified 5 separate variants absent from the general population, 9 of which were established to be de novo events, and 4 of which had occurred recurrently. Taken together, this provided strong genetic evidence for heterozygous variants in *LMNB1* and *LNMB2* as a cause of microcephaly.

### Clinical phenotype of *LMNB1/2* individuals

**Fig. 2:**
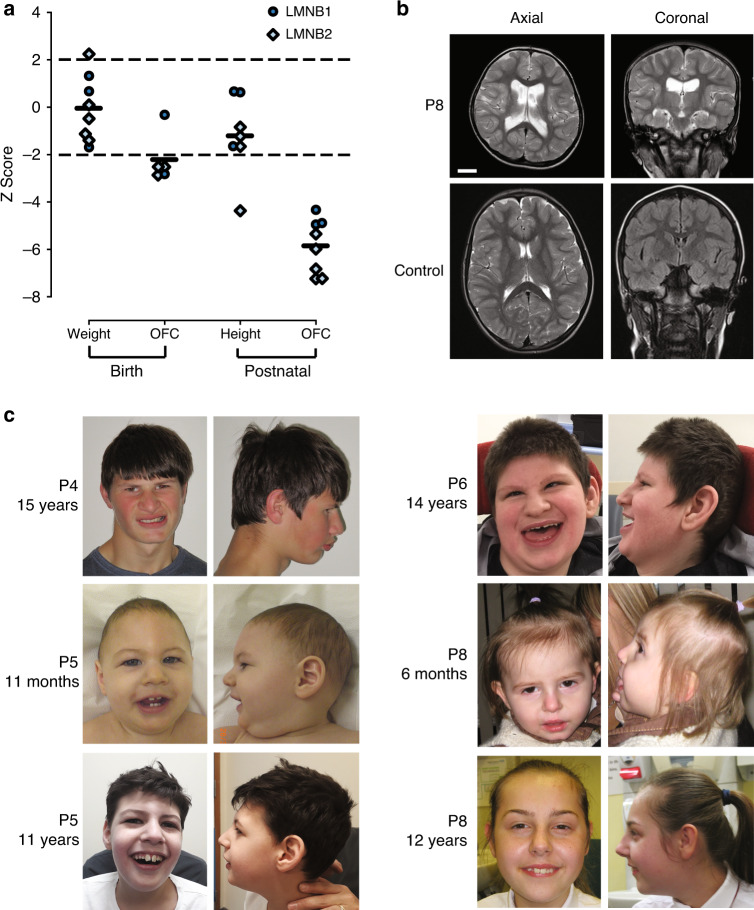
Pathogenic *LMNB1* or *LMNB2* variants in patients with primary microcephaly. (**a**) Growth parameters of individuals with pathogenic *LMNB1/2* variants plotted as *Z*-scores (standard deviations from the age and sex-matched mean). Black bars, mean *Z*-score. Dashed lines, 95% confidence interval for general population. (**b**) Neuroimaging demonstrates reduced cortical size with simplified gyri. Ventricular dilatation reflects accompanying global reduction in white matter. Comparison with age-matched control. Scale bar 2 cm. Axial images, T2-weighted. Coronal, T2 (P8), fluid-attenuated inversion recovery (FLAIR) (control). (**c**) Photographs of individuals P4, P5, P6, and P8. Written consent obtained from families for photography. *OFC* occipital frontal circumference.

All LMNB1/2 cases had severe microcephaly (OFC −5.85 ± 1.14 SD, Fig. 2a, Table S5), evident from birth in all but one case. No intrauterine growth retardation was evident, with birth weights within normal range. Postnatally, height also remained within normal ranges for most cases. Global developmental delay of varying severity was evident and seizures present in four cases (Tables S1, S3). Major malformations were not reported. Neuroimaging demonstrated a structurally normal brain, without evidence of abnormal neuronal migration (Fig. 2b). In two cases gyral simplification was noted accompanied by global reduction in white matter volume alongside increased extra-axial spaces and enlarged ventricles. In P8, where serial scans were available, this was nonprogressive. Facially, aside from a sloping forehead in some individuals, a syndromic diagnosis was not evident, nor was a consistent facial gestalt observed (Fig. 2c).

### Predicted consequences for lamin B1/B2 polymer assembly

While no LMNB1/2 protein structures are available that cover regions where the variants occur, all mutated residues are conserved with LMNA. Therefore, LMNA crystal structures could be used to model the substitutions. Each variant alters a residue in a coiled-coil segment of the α-helical rod domain of LMNB1/2 (Fig. 1a). LMNB1 Lys33 and LMNB2 Asn54 lie at the intradimer interface of coil 1A, LMNB1 Arg90 is at the interdimer interface of coil 1B, and LMNB2 Glu398 is at the intradimer interface of coil 2B. Substitution or deletion of these residues would therefore likely interfere with dimer or filament assembly. Notably, both p.Lys33Glu and p.Glu398Lys result in mutated residues with opposite electrostatic charges to the wild-type residue. LMNA Glu383, corresponding to LMNB2 Glu398, is established to separate the two lamin chains of the dimer at the end of the rod domain, where it repels Glu383 from the other chain.^9^ Therefore the heterozygous LMNB2 p.Glu398Lys substitution would result in a subset of mismatched dimers in which, instead, interaction of the two chains would be aberrantly stabilized, with a positively charged mutant lysine residue forming ionic interactions with the negatively charged wild-type glutamate. Molecular modeling with FoldX predicts this substitution to strongly stabilize the interdimer interaction (ΔΔG of −2.0 kcal/mol, Table S6), in keeping with a proposed stabilizing mechanism for pathogenic LMNA variants.^10^

Evidence for pathogenicity can also be drawn from the observation that all mutated codons are conserved in lamin A/C paralogs. Indeed, three of the LMNB variants (Lys33, Asn54, Arg90) correspond to residues in LMNA that are altered in lamin A/C associated disorders.^11^ As substitution of these sites in LMNA are disease causing, this would also be expected to be the case in LMNB1/B2. Furthermore, LMNB1 p.Lys33del has already been modeled in the ancestral *C.elegans LMN* (a protein most homologous to B-type lamins^1^) where it disrupts protofilament assembly in vitro and causes nuclear aggregates of lamin in vivo.^12^

Lastly, we examined the cellular distribution of GFP-lamin B fusion proteins containing microcephaly variants, in a similar manner to that done previously for LMNA variants.^13^ Cells expressing the LMNB1/B2 variants frequently contained nuclear aggregates and/or significantly altered nuclear morphology in comparison to cells expressing wild-type GFP-LMNB1/2 (Fig. S2, Tables S8 and S9). While GFP-LMNB1/2 proteins were expressed above endogenous levels, both aggregates and altered nuclear shape have previously been reported for pathogenic lamin A/C variants, both when overexpressed^13^ and at endogenous levels,^12,14^ leading us to conclude that lamin B variants may also disrupt lamin assembly in cells.

## DISCUSSION

### LMNB primary microcephaly: a new laminopathy

Here we identify recurrent and de novo heterozygous variants in *LMNB1* and *LMNB2* in eight microcephaly patients from the DDD cohort. The additional five cases ascertained from 100kGP on the basis of de novo or recurrent variants in LMNB1/2 also had microcephaly, confirming the phenotype–genotype link. A severe nonsyndromic microcephaly without other malformations was consistently present. In keeping with primary microcephaly, all but one case had a reduced OFC evident at birth, and neuroimaging demonstrated a structurally normal small brain with/without a simplified gyral pattern. Dilated ventricles and increased axial spaces have been previously documented,^15,16^ as have seizures in a minority of primary microcephaly cases. Primary microcephaly is usually accompanied by mild/moderate intellectual disability, although for LMNB1/2 global developmental delay varied substantially between cases, classified as severe in four of eight DDD cases, with speech not attained in three older children.

The LMNB1/B2 microcephaly phenotype is distinct from previously described lamin A/C laminopathies, pointing to different cellular and developmental roles for lamin B proteins, despite all lamins acting as major structural components of the nuclear envelope. The human microcephaly is in keeping with the previously reported mouse knockout model for *Lmnb1* in which cerebral cortical size was markedly reduced.^17^ However, in both the *Lmnb1*^*−/−*^ and *Lmnb2*^*−/−*^ mice, (where brain size was not reduced at birth), there were neuronal migration defects resulting in abnormal layering of the cerebral cortex,^18^ suggesting potential discordance with the human phenotype and neuroimaging findings. This may be accounted for however by a different mechanism in human cases where variants are heterozygous rather than biallelic null. The pathogenic variants reported here are most likely dominant negative rather than haploinsufficient given that microcephaly is not present in the Lmnb1^+/−^ mouse. The recurrent nature of the pathogenic variants at specific residues and absence of truncating variants favors gain of function or dominant negative mechanisms. Furthermore, substitutions in corresponding residues in lamin A/C do not destabilize the protein, rather disrupt lamin filament formation,^19^ a common scenario by which point variants in multimeric proteins can act in a dominant negative manner to perturb macromolecular complexes.^20^

LMNB1 and LMNB2 have overlapping but distinctive cellular and developmental roles.^1^ So it is striking that pathogenic variants in both cause primary microcephaly, suggesting an etiology in which a shared function is disrupted. Many primary microcephaly proteins are involved in mitosis, and often encode centrosomal or spindle components that when mutated result in altered spindle orientations disrupting symmetric cell division dynamics in neural progenitors.^7^ This reduces neuronal cell number generated during neurogenesis. B-type lamins associate with the mitotic spindle^21^ with defects in spindle orientation in neuronal precursor cells reported in *Lmnb1* and *Lmnb2* knockout mice,^17^ suggesting this could be the mechanism underlying microcephaly. Other mitotic processes may also be perturbed, given the involvement of LMNs in nuclear envelope reassembly. However, the potential for *LMNB1/2* variants to alter the physical properties of lamin dimers and filaments (Fig. 1c) should also be considered, given that several of the variants are seen to impact on cellular assembly of nuclear lamina, and that the equivalent pathogenic LMNA variant to LMNB1 p.Lys33del results in fragile nuclei more prone to mechanical deformation.^22^ Such fragility in neuronal precursors would enhance susceptibility to mechanical stresses of interkinetic nuclear migration and neuronal migration and lead to increased cell death during brain development.^23^

Notably, several genes encoding components of the nuclear pore have also been implicated in microcephaly. While nuclear pores are also important components of the nuclear envelope, these are generally clinically distinct disorders, often associated with nephrotic syndrome (Galloway–Mowat syndrome^24,25^) or with a progressive microcephaly/encephalopathy.^26,27^ As well, nuclear–cytoplasmic transport has been demonstrated to be impaired,^27^ so the microcephaly in nuclear pore disorders could have a different mechanistic basis.

In conclusion, our findings establish heterozygous variants in *LMNB1* and *LMNB2* as causes of primary microcephaly, implicating the nuclear lamina in its etiology and defining a novel form of laminopathy. Future in vitro and in vivo studies will be important to provide further evidence for causality of the variants reported here, and to shed light on how they cause a laminopathy distinct from those previously described.

## Supplementary information

Supplementary information
