# Supplementary material for: Heterozygous lamin B1 and lamin B2 variants cause primary microcephaly and define a novel laminopathy
Source: Genet Med. 2020 Oct 9;23(2):408–14. doi: 10.1038/s41436-020-00980-3 (PMC7862057; doi:10.1038/s41436-020-00980-3)
Supplement: Supplementary file 1 — Supplementary information [file 41436_2020_980_MOESM1_ESM.pdf]

Heterozygous Lamin B1 and Lamin B2 Mutations cause Primary Microcephaly and Define a Novel Laminopathy

SUPPLEMENTARY INFORMATION

Supplementary Tables 2

Table S1: Clinical features of DDD subjects with *LMNB1* or *LMNB2* mutations. 2

Table S2 Additional phenotype information on DDD *LMNB1/B2* subjects 3

Table S3: Clinical features of subjects with de novo *LMNB1* or *LMNB2* mutations in 100kGE study 4

Table S4: *In silico* predictions of *LMNB1* and *LMNB2* variant consequences 5

Table S5: Anthropometric measurements of patients with *LMNB1* or *LMNB2* mutations. .. 6

Table S6: Fold X predictions for missense variants identified in this study......................... 7

Table S7: Variants found in other microcephaly genes 7

Table S8: GFP-Lamin nuclear aggregate quantification.. 8

Table S9: Nuclear roundness ratio measurements based on nuclear area and long axis measurements.. 8

Supplementary Figures 9

Figure S1: Capillary sequence traces for P7 and mother. 9

Figure S2: Expression of microcephaly variants cause GFP-Lamin B nuclear aggregate formation and altered nuclear morphology... 10

Supplementary Methods 11

Prevalence of primary and secondary microcephaly in DDD cohort................................ 11

De novo variant calling criteria.. 11

100,000 genomes project – identification of further *LMNB* variants 11

Exclusion of variants in known microcephaly/microcephalic dwarfism genes................... 11

Structural modelling.. 12

Cell culture and generation of stable cell lines 12

Immunofluorescence microscopy and analysis... 12

Statistics.. 13

References 13

Supplementary Tables

Table S1: Clinical features of DDD subjects with *LMNB1* or *LMNB2* mutations.

Patient	P1	P2	P3	P4	P5	P6	P7	P8
**Sex**	Male	Female	Female	Male	Male	Male	Female	Female
**Gene**	*LMNB1*	*LMNB1*	*LMNB1*	*LMNB2*	*LMNB2*	*LMNB2*	*LMNB2*	*LMNB2*
**Variant**	c.97A>G p.K33E	c.97_99del p.K33del	c.97_99del p.K33del	c.1192G>A p.E398K	c.1192G>A p.E398K	c.1192G>A p.E398K	c.1192G>A p.E398K	c.160A>C p.N54H
**De novo**	Y	Y	Unknown	Y	Y	Unknown	N	Y
**Age**	1mo	18yrs	8yrs	15yrs	11 years	14yrs 3 mo	10 yrs 10mo	12yrs 3mo
**Microcephaly**	Y	Y	Y	Y	Y	Y	Y	Y
**Cranial MRI**	Simplified gyri large ventricles, Increased extra-axial space	Normal	Normal	Normal	Normal	Normal	Normal	Simplified gyri. Enlarged ventricles, Increased extra-axial space. Non progressive.
**Other CNS**	Seizures	Dyspraxia			Fine tremulous movements of hands, clenched fists, limb hypertonia and brisk reflexes, normalized at 2yrs.	Tremor, Absence seizures from 5yr, 1-2 per year. Normal EEG. Increased leg tone.	Hypotonia	
**Developmental Delay**	Severe	Mild to moderate	Moderate	Severe	Severe	Severe	Moderately Severe	Moderate
**Age of Walking**	Not achieved	15 months	2.5-3 years	3 years	2yrs 10m	Not achieved	6 years	20 months
**Speech**	Not achieved	3-4 years	Occassional words at 7 uses makaton	Not achieved	Not achieved Vocalises only uses communication device.	Not achieved. Uses communication device	20 months	Unknown

Table S2 Additional phenotype information on DDD *LMNB1/B2* subjects

Patient	P1	P2	P3	P4	P5	P6	P7	P8
**Sex**	Male	Female	Female	Male	Male	Male	Female	Female
**Gene**	*LMNB1*	*LMNB1*	*LMNB1*	*LMNB2*	*LMNB2*	*LMNB2*	*LMNB2*	*LMNB2*
**Variant**	c.97A>G p.K33E	c.97_99del p.K33del	c.97_99del p.K33del	c.1192G>A p.E398K	c.1192G>A p.E398K	c.1192G>A p.E398K	c.1192G>A p.E398K	c.160A>C p.N54H
**Facial Appearance**	Up-slanting palpebral fissures; Right anterior helical ear pit	Sloping forehead		Sloping forehead; Wide mouth; Prominent nasal bridge	Epicanthus inversus; Ptosis; Long palpebral fissures, prominent nasal bridge, metopic ridge, prominent central incisors	Deep set eyes, wide mouth, wide spaced teeth, brachycephaly	Prominent epicanthal folds; Long palpebral fissures; low set ears	Sloping forehead; high nasal bridge; retrognathia. Large earlobes.
**Cardiac**			Atrial septal defect	Small VSD - closed spontaneously				
**Skeletal**		Severe Kyphosis.	2nd toe overrides 3rd bilaterally.	5th finger clinodactyly; Short, tapering fingers	Trigonocephaly. Bilateral Metatarsus adductus. Long hallux	Hip subluxation	Short broad fingers	5th finger Clinodactyly
**Skin**		Eczema		Normal	Eczema		Normal	Cutis marmorata on arms and legs
**Family history**	Nil; one older sibling		> 3 previous pregnancy losses. ID[1] in mother and two sibs	Nil; 2 older siblings	Maternal uncle Pearson syndrome died 5y age, Cousin DWV[2] died 4d old		2 previous pregnancy losses. 1 older male sib	
**Other**	Recurrent lower respiratory tract infections, gastrostomy. ?pan-hypopit.	Sleep disturbance, Hyperhydrosis. Restrictive eating pattern, Autistic features. Recurrent urinary symptoms	Hypothyroidism.	Micropenis, Undescended testes, joint hypermobility; unusual abducted scapulae	Severe constipation, severe hayfever Sleep disturbance, slow feeding as a baby (2p14 dup pat likely benign)	Teeth grinding. Orchidopexy, Constipation. Cannot lie flat	Drooling. Astigmatism	Large earlobes. Stippled retina

ID[1], intellectual disability.[2]Dandy Walker variant

Table S3: Clinical features of subjects with de novo *LMNB1* or *LMNB2* mutations in 100kGE study

Patient	P9	P10	P11	P12	P13
**Sex**	Male	Male	Female	Male	Female
**Gene**	*LMNB1*	*LMNB1*	*LMNB1*	*LMNB2*	*LMNB1*
**Variant**	c.97A>G p.K33E	c.269G>C p.R90P	c.97A>G p.K33E	c.1192G>A p.E398K	c.269G>C p.R90P
**De novo**	Y	Y	Y	Y	Unknown, mother affected
**Age Category (years)**	5-10	15-20	5-10	0-5	10-15
**Microcephaly**	Y	Y	Y	Y	Y
**Other CNS**	Febrile Seizures		Seizures, Hypoplasia/aplasia corpus callosum		
**Developmental Delay**	GDD	Mild/Mod GDD	Mild/Mod GDD	GDD	Intellectual Disability
**Speech**	Absent speech	Delayed speech and language development			
**Other**	Failure to thrive, poor feeding. Constipation. Stereotypical hand movements. Long palpebral fissures, hypertelorism, depressed broad nasal bridge. Pointed chin.	Hydronephrosis. Failure to thrive Hypothyroidism		Short stature. Hearing impairment	

GDD, global developmental delay; P10-P13, phenotype information derived from encoded HPO terms in the Genomes England dataset

Table S4: *In silico* predictions of *LMNB1* and *LMNB2* variant consequences

Genomic Coordinate (GRCh37)	REF	ALT	Gene	Observations	cDNA Consequence	Protein Consequence	PolyPhen2 (HVAR)	SIFT	Mutation Taster	CADD PHRED (GRCh37-v1.6)	Ensembl Transcript
5:126113294	GAGA	G	*LMNB1*	2	c.97_99del	p.Lys33del	N/A	N/A	Disease causing (0.999)	20.8	ENST00000261366.10
5:126113297	A	G	*LMNB1*	3	c.97A>G	p.Lys33Glu	probably damaging (0.999)	deleterious (0)	Disease causing (0.999)	26.9	ENST00000261366.10
5:126113469	G	C	*LMNB1*	2	c.269G>C	p.Arg90Pro	probably damaging (1.0)	deleterious (0)	Disease causing (0.999)	29.3	ENST00000261366.10
19:2456772	T	G	*LMNB2*	1	c.160A>C	p.Asn54His	probably damaging (1.0)	deleterious (0)	Disease causing (0.999)	26.7	ENST00000325327.3
19:2434303	C	T	*LMNB2*	5	c.1192G>A	p.Glu398Lys	probably damaging (0.999)	deleterious (0)	Disease causing (0.999)	31.0	ENST00000325327.3

Table S5: Anthropometric measurements of patients with *LMNB1* or *LMNB2* mutations.

		Birth			Postnatal			
Patient	Gene	Gestation /weeks	Weight /kg (SD)	OFC /cm (SD)	Age at Measurement	Weight /kg (SD)	OFC /cm (SD)	Height /cm (SD)
P1	*LMNB1*	39	3.66 (0.67)	31 (-2.82)	1 month	4.30 (-0.33)	31.5 (-4.89)	56.0 (0.63)
P2	*LMNB1*	38	3.57 (1.32)	33 (-0.32)	10 years 11 months	37.30 (0.23)	48.5 (-4.33)	148.2 (0.66)
P3	*LMNB1*	40	2.66 (-1.69)	N/A	12 months	6.38 (-4.13)	41.0 (-4.95)	71.0 (-1.65)
P4	*LMNB2*	40	3.32 (-0.48)	32 (-2.52)	15 years 5 months	48.40 (-1.04)	47.4 (-5.35)	157.9 (-1.66)
P5	*LMNB2*	40	2.89 (-1.40)	32 (-2.52)	27 months	9.94 (-2.68)	41.5 (-6.83)	87 (-0. 84)
P6	*LMNB2*	42	3.60 (0.09)	N/A	14yrs 3 months	39.8 (-1.37)	46 (-5.99)	N/A
P7	*LMNB2*	41	4.48 (2.24)	N/A	10yrs 5 months	17.1 (-4.67)	43.5 (-7.24)[1]	112 (-4.37)
P8	*LMNB2*	40	2.90 (-1.13)	31 (-2.87)	12 years 3 months	36.7 (0.79)	45 (-7.23)	142.8 (-1.23)

SD, Z-scores calculated using LMS growth, British 1990 dataset. [1]OFC measurement from 6y10months.

Table S6: Fold X predictions for missense variants identified in this study.

Protein	Variant	ΔΔG (kcal/mol)
LMNB1	p.Lys33Glu	0.6
LMNB2	p.Asn54His	0.2
LMNB1	p.Arg90Pro	4.0
LMNB2	p.Glu398Lys	-2.0

Table S7: Variants found in other microcephaly genes

Patient	Gene	Expected Inheritance	Observed Genotype	Variant	dbSNP ID	Max Allele Frequency in gnomAD (v2.1)
P6	*CDT1*	Recessive	Heterozygous (inheritance unknown)	c.1354G>C; p.Glu452Gln	rs1309417334	$2.37e^{-4}$
P13	*PHC1*	Recessive	Heterozygous (inheritance unknown)	c.1540_1551del; p.Lys514_Ala517del	rs570712396	0.0024

Rare variants (< 0.5% in gnomAD v2.1) predicted to alter coding sequence or splice junctions in canonical transcripts and segregating with the disease phenotype within families were identified. Analysis for the following genes associated with microcephaly or microcephalic osteodysplastic dwarfism spectrum disorders: *ANKRD11, ARCN1, ASPM, ATR, ATRIP, ATRX, BLM, CASC5, KNL1, CDC45, CDC6, CDK5RAP2, CDKN1C, CDT1, CENPE, CENPF, CENPJ, CEP135, CEP152, CEP63, DNA2, DNMT3A, DONSON, DPP6, GMNN, IGF1, IGF1R, KMT2A, LARP7, LIG4, MCPH1, NDE1, ORC1, ORC4, ORC6, PCNT, PHC1, PLK4, PNKP, POC1A, POLE, RBBP8, RNU4ATAC, SMARCAL1, STIL, TOP3A, TRAIP, TUBGCP6, WDR4, WDR62, XRCC4*.

Table S8: GFP-Lamin nuclear aggregate quantification. Statistics are given for values from three independent experiments, represented graphically in Figure S2B.

Gene	Variant	Mean	Standard Deviation
LMNB1	**WT**	1.210	1.358
LMNB1	**p.Arg90Pro**	41.73	4.733
LMNB1	**p.Lys33Glu**	10.54	3.577
LMNB1	**p.Lys33del**	16.49	2.680
LMNB2	**WT**	0.3003	0.5201
LMNB2	**p.Asn54His**	93.46	2.486
LMNB2	**p.Glu398Lys**	95.76	1.612

Table S9: Nuclear roundness ratio measurements based on nuclear area and long axis measurements. Statistics are given for values from three independent experiments, represented graphically in Figure S2C.

Gene	Variant	Mean	Standard Deviation
LMNB1	**WT**	0.72	0.0021
LMNB1	**p.Arg90Pro**	0.69	0.0059
LMNB1	**p.Lys33Glu**	0.65	0.0085
LMNB1	**p.Lys33del**	0.65	0.0155
LMNB2	**WT**	0.73	0.0075
LMNB2	**p.Asn54His**	0.64	0.0066
LMNB2	**p.Glu398Lys**	0.60	0.0038

Supplementary Figures

Figure S1: Capillary sequence traces for P7 and mother. Exome sequencing data showed 6 variant reads out of a total 28 reads covering this site in the mother of P7, suggesting mosaicism. Confirmatory Sanger sequencing shows a small peak for the *LMNB2* c.1192G>A variant allele in blood derived DNA from the mother of P7, consistent with mosaicism.

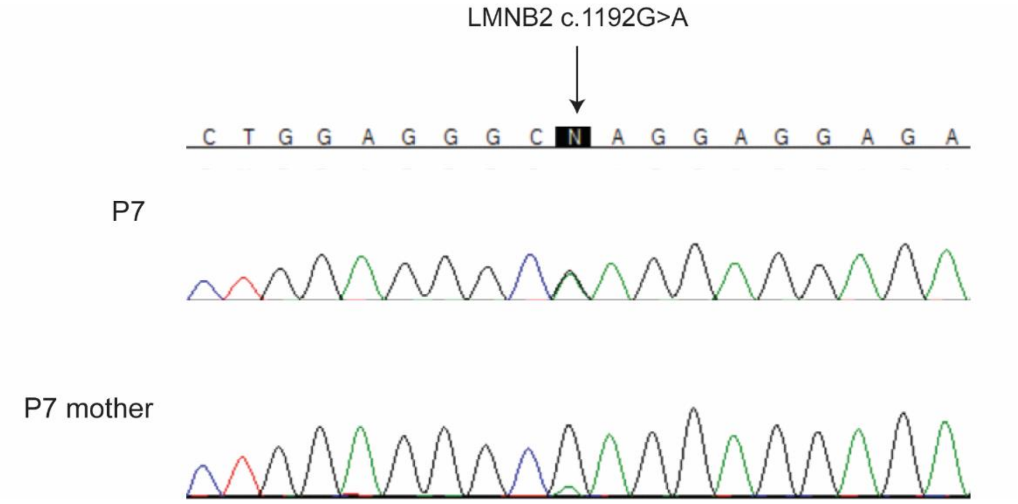

Figure S2: Expression of microcephaly variants cause GFP-Lamin B nuclear aggregate formation and altered nuclear morphology. (A) Representative images of RPE1 clonal cell lines with doxycycline-induced expression of GFP-Lamin B1 and B2 wild-type and microcephaly variant proteins for 48 hrs. RPE1 cells overexpressing lamin B1 and B2 variant proteins (LMNB1-K33E/K33del/R90P or LMNB2-N54H/E398K) demonstrated irregularities of nuclear shape and formation of GFP-Lamin B nuclear aggregates (examples of cells with aggregates of varying size and density, yellow arrowheads). Scale bar, 10 µm. (B) Quantification of nuclear aggregates in GFP-Lamin B1 and B2 expressing RPE1 cells. Variant proteins are more likely to form aggregates compared to wild type protein, with LMNB2-N54H and LMNB2-E398K having a more profound effect on frequency of aggregates (experiments = 3, n>100 per expt, error bars, s.d.) (C & D) Analysis of nuclear shape from experiment in (A). (C) Dot plots of nuclear roundness ratios (derived from nuclear area and long axis measurements). RPE1 cells expressing lamin B1 and B2 variant proteins have significantly decreased roundness ratio compared to wild type counterparts. For cells expressing GFP-LMNB1-R90P the difference is less substantial but remains statistically significant. Each point represents the roundness measurement of an individual cell with 3 independent experiments depicted in green, blue or red dots, n>1430 cells per experiment. Mean values per experiment are provided as filled colored circles. Bars represent median and quartile ranges for all cells combined across experiments. (D) Relative frequency distribution of the roundness ratio for the cells represented in (C). RPE1 cells expressing mutant lamin B1 and B2 proteins had a wide range of nuclear roundness ratios, with the distribution shifted towards the lower values compared to cells expressing the wild type counterparts. All P values were calculated by one-way ANOVA with Dunnett's posthoc correction test (****P ≤ 0.0001; ***P ≤ 0.001; **P ≤ 0.01; *P ≤ 0.05).

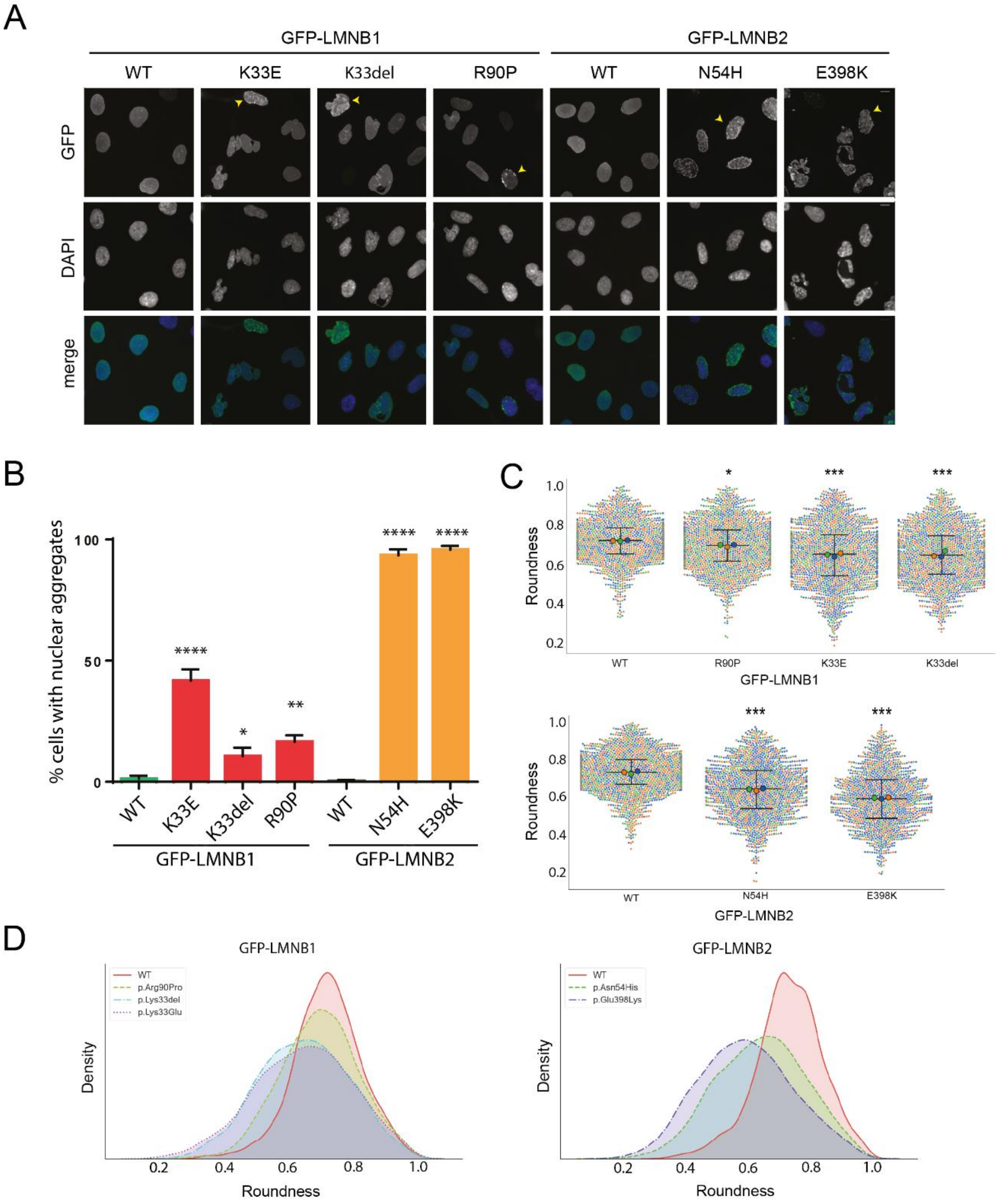

Supplementary Methods

Prevalence of primary and secondary microcephaly in DDD cohort

The proportion of microcephaly cases with primary or secondary microcephaly was estimated from cases where birth OFC was available (n=349), using birth OFC of ≤ -2sd[1] as a criteria for prenatal-onset microcephaly to distinguish primary microcephaly from secondary (postnatal-onset) microcephaly.

De novo variant calling criteria

For the *de novo* inheritance model, DDD variants were required to have a minimum depth of 8 reads in each individual in the parent-child trio with 1:4 minimum ratio of alternative to reference reads in the child (minimum observed depth and ratio for *LMNB1/2 de novo* variants identified in this study was 19 and 0.42 respectively), maximum 1:20 ratio in parental reads and minimum PHRED-like genotype quality scores of 20 in each individual. Variants were excluded if present in any gnomAD population at a frequency ≥ 0.01% or if the variant site had more than two alternate alleles. Variants were also removed if not classified as either protein altering or as splice acceptor/donor variants by Ensembl's Variant Effect Predictor[2].

100,000 genomes project – identification of further *LMNB* variants

Further *LMNB1/B2* variants were identified from the 100,000 genomes project (100kGP, Genomics England), under the auspices of the Rare Disease GeCIP, utilising this as an additional patient cohort for study validation, with variants and HPO terms extracted from the Genomics England research environment. This was performed without specifying phenotype, with all *de novo* variants in LMNB1/B2 ascertained. Subsequently, data were re-queried to identify further occurrences of the *de novo* variants found in either DDD or 100kGP datasets.

Exclusion of variants in known microcephaly/microcephalic dwarfism genes

For each individual reported in this study we analysed sequence data to identify potentially pathogenic variants in the following genes previously associated with microcephaly or microcephalic dwarfism spectrum disorders: *ANKRD11, ARCN1, ASPM, ATR, ATRIP, ATRX, BLM, CASC5, KNL1, CDC45, CDC6, CDK5RAP2, CDKN1C, CDT1, CENPE, CENPF, CENPJ, CEP135, CEP152, CEP63, DNA2, DNMT3A, DONSON, DPP6, GMNN, IGF1, IGF1R, KMT2A, LARP7, LIG4, MCPH1, NDE1, ORC1, ORC4, ORC6, PCNT, PHC1, PLK4, PNKP, POC1A, POLE, RBBP8, RNU4ATAC, SMARCAL1, STIL, TOP3A, TRAIP, TUBGCP6, WDR4, WDR62, XRCC4*. Variants were filtered using VASE (v0.2.4, https://github.com/david-a-parry/vase) to identify rare (< 0.5% allele frequency in gnomAD

v2.1) alleles predicted to be protein altering or splice acceptor/donor variants in canonical transcripts and segregating consistent with recessive or dominant inheritance within families.

Structural modelling

FoldX v4[3] was run using default parameters on homodimeric (PDB ID: 1X8Y) and homotetrameric (PDB ID: 6JLB) crystal structures of LMNA covering the mutated residues. The 'BuildModel' function of FoldX was used to repair structures, and ten replicates were performed for each mutation. Missense variants were modelled separately for each chain in the homodimer and each of the four chains in the homotetramer, with average $\Delta\Delta G$ values over all genes calculated.

Cell culture and generation of stable cell lines

hTERT RPE-1 cells obtained from the American Type Culture Collection (CRL-4000) were maintained in DMEM:F12, 10% FCS, 100 U/ml penicillin and 100 µg/ml streptomycin at 37 °C with 5% CO2. All cell lines were routinely tested for mycoplasma.

hTERT RPE1 cells stably expressing GFP-LMNB1-WT, GFP-LMNB1-K33E, GFP-LMNB1-K33del, GFP-LMNB1-R90P, GFP-LMNB2-WT, GFP-LMNB2-N54H and GFP-LMNB2-E398K were generated as follows. Wild-type full length transcripts of LMNB1 and LMNB2 were amplified by PCR from human primary fibroblast cDNA using PCR primers with flanking attB sites (primer sequences available on request) and the PCR product recombined into pDONR221 vector by a BP reaction (Gateway cloning, Thermo Fischer Scientific). LMNB1/B2 variants were then introduced by site directed mutagenesis (Agilent) and shuttled into pXLONE-GFP-DEST[4] (a vector derived from XLone-GFP, a gift from Xiaojun Lian (Addgene plasmid #96930; http://n2t.net/addgene:96930 ; RRID:Addgene_96930).

pXLONE-GFP-LMNB1/2 constructs and mPB (a piggybac transposase expression construct, gift Prof Richard Meehan lab) were co-transfected into hTERT RPE-1 cells. Stable integrants were selected using 20 µg/ml blasticidin and clonal cell lines were derived. To achieve as close to endogenous levels as possible, clones were selected for characterization on the basis of the clone with lowest GFP-lamin expression levels, and a low concentration of doxycycline (50 ng/ml) was also used to induce protein expression.

Immunofluorescence microscopy and analysis

hTERT RPE-1 cells were grown on untreated coverslips, fixed with 4% paraformaldehyde in PHEM (25 mM HEPES-NaOH, pH 6.8, 100 mM EGTA, 60 mM PIPES, 2 mM MgCl2) for 20 min at 37°C. After fixation cells were permeabilized by treatment in 0.2% Triton X-100 in PHEM for 2 min, then incubated with 2 µg/ml DAPI in PBS for 60 mins. Epifluorescent images were acquired using an Axioplan 2 wide-field fluorescence microscope (Zeiss) with a

Prime BSI camera (Photometrics). Images were captured with a 40× Plan-APOCHROMAT (1.4 NA) objective using Micro-Manager software (http://open-imaging.com/). For quantitative nuclear shape analyses, images were analyzed using a custom-written ImageJ based script to trace the outline of DAPI stained nuclei and compute the nuclear roundness ratio: $4*\text{area}/(\pi \times [\text{Major axis}]^2)$. The roundness ratio reaches a maximum value of 1 for a circle and decreases with increasingly asymmetric and irregular nuclear shapes.

Statistics

Experimental sample sizes were chosen based on standard practices in the field with experiments replicated at least 3 times. No statistical method was used to pre-determine sample size. Statistical analysis was performed in R and Prism v.6 (Graphpad). One-way ANOVA was used to test for statistical differences between group means for 3 independent experiments, under the assumption of normal distribution of variation between experiments. Dunnett's posthoc test was used to perform comparison of multiple conditions to a control (WT) with correction for multiple testing.

References

1. Shaheen R, Maddirevula S, Ewida N, et al. Genomic and phenotypic delineation of congenital microcephaly. *Genet Med*. September 2018:1. doi:10.1038/s41436-018-0140-3

2. McLaren W, Gil L, Hunt SE, et al. The Ensembl Variant Effect Predictor. *Genome Biol*. 2016;17(1):122. doi:10.1186/s13059-016-0974-4

3. Schymkowitz J, Borg J, Stricher F, Nys R, Rousseau F, Serrano L. The FoldX web server: an online force field. *Nucleic Acids Res*. 2005;33(Web Server):W382-W388. doi:10.1093/nar/gki387

4. Randolph LN, Bao X, Zhou C, Lian X. An all-in-one, Tet-On 3G inducible PiggyBac system for human pluripotent stem cells and derivatives. *Sci Rep*. 2017;7(1). doi:10.1038/s41598-017-01684-6
